# 662. Trapezoid Advancement Flaps. Treatment of Severe Upper Extremity Burn Contractures Limiting the Risk of Recurrence

**DOI:** 10.1093/jbcr/irag033.528

**Published:** 2026-04-06

**Authors:** Tertius H J Venter

**Affiliations:** Mercy Ships, Amsterdam, Noord-Holland

## Abstract

**Introduction:**

Severe postburn contractures cause devastating functional loss, particularly in low-middle-income countries where > 90% of burns occur. Traditional reconstruction faces high recurrence rates and graft breakdown. We developed a novel technique that leverages the observation that most contractures exhibit healthy skin on one side and scarring on the other.

**Methods:**

A retrospective analysis of 27 patients (mean age 16.4 years) treated for elbow/axilla contractures. The technique involves excising the main contracture band, creating a trapezoid flap from the healthy skin side, advancing it over the joint surface, and positioning skin grafts proximal and distal to the flexion creases. Primary outcomes were assessed: range of motion (ROM) and complications.

**Results:**

Twenty-nine elbow and 16 axilla contractures treated - more than one procedure in some patients. Shoulder ROM improved significantly from 111.0° to 149.4° abduction-adduction (mean improvement 38.4°, p<.05). Elbow ROM improved from 76.6° to 108.6° flexion-extension (improvement 33.1°, p<.05). Elbow contracture reduced from 60.5° to 18.5° (p<.05). One patient required revision for compartment syndrome; ^2^no skin loss or graft failure occurred. Average follow-up: 3 months.

**Conclusions:**

Trapezoid advancement flaps provide reliable functional improvement with minimal complications by positioning healthy tissue over joint surfaces and grafts away from high-stress areas. This technique addresses recurrence through biomechanically sound tissue placement, making it ideal for resource-limited settings where contracture burden is highest. The approach allows complete scar excision while providing durable coverage, representing a significant advancement in burn contracture reconstruction with excellent short-term outcomes and low complication rates.

**Applicability of Research to Practice:**

The technique essentially transforms a complex reconstructive challenge into a more predictable procedure with better long-term outcomes, making quality burn contracture care accessible in diverse environments.

**Funding for the study:**

N/A.

